# Clinical Management of Pediatric Hemosuccus Pancreaticus: Experience Based on a Case Report and Mini Literature Review

**DOI:** 10.1155/crgm/4250947

**Published:** 2026-10-26

**Authors:** Donghao Tian, Jialu Fu, Hui Yu, Qiang Liu, Yuekui Luo, Baijun Zheng, Ya Gao

**Affiliations:** ^1^ Department of Pediatric Surgery The Second Affiliated Hospital Xi’an Jiaotong University No 157, Xi Wu Road Xi’an 710004 China xjtu.edu.cn; ^2^ Department of Radiology The Second Affiliated Hospital Xi’an Jiaotong University No 157, Xi Wu Road Xi’an 710004 China xjtu.edu.cn; ^3^ Department of Ultrasound Medicine The Second Affiliated Hospital Xi’an Jiaotong University No 157, Xi Wu Road Xi’an 710004 China xjtu.edu.cn

**Keywords:** gastrointestinal bleeding, interventional therapy, pancreatic pseudocyst, peripancreatic pseudoaneurysm

## Abstract

**Objective:**

Hemosuccus pancreaticus (HP) rarely happens in children, and early diagnosis poses significant challenges. It aims to elucidate the clinical characteristics and management of pediatric HP.

**Methods:**

To retrospectively analyzes a pediatric HP case admitted to our department, along with a narrative literature review.

**Results:**

A 13‐year‐old boy presented intermittent epigastric pain with gastrointestinal bleeding (GIB), who previously suffered from chronic pancreatitis. The emergency gastroscopy showed fresh blood overflow from duodenal papilla, and CT angiography (CTA) revealed a pancreatic tail pseudocyst with splenic artery (SpA) involved and occurring pseudoaneurysm, which suggested HP. Twice interventional therapy yielded hemostatic effect while appeared prone to recurrence, laparoscopic resection of pancreatic pseudocyst, SpA pseudoaneurysm and partial splenectomy was performed. The child did not experience recurrence with follow‐up for 2 years. Another 9 children with the age of 3–18 years were reported; 6 cases were diagnosed via CT arteriography and successfully interventional embolized, and 2 cases were diagnosed and treated using endoscopic ultrasonography (EUS).

**Conclusion:**

Pediatric HP is attributed to peripancreatic pseudoaneurysm ruptured into pancreatic duct to duodenum, secondary to pancreatitis, abdominal surgery, or blunt abdominal trauma. Intermittent epigastric pain, GIB, and elevated blood amylase are common clinical features. Endoscopy often missed diagnosis and even requires repeated examination. CTA is recommended for HP diagnosis. Interventional therapy is the first choice but leading to complications, especially patients with failed interventional, ineffective hemostasis or hemodynamic instability can be treated with laparoscopic surgery. Sometimes, the EUS is alternative. Generally, the children if diagnosed in time present good prognosis.

**Highlights**
• What is known
◦ Hemosuccus pancreaticus (HP) rarely happens in children, and reports are few case studies; therefore, early identification is significantly difficult and poses challenges to standardized treatment.
◦ Delayed diagnosis of HP leads to progressively worsening anemia, potentially life‐threatening acute upper gastrointestinal bleeding (GIB), which often poses a lethal risk.
• What is new:
◦ This study comprehensively elucidated the clinical characteristics, diagnosis, and therapy of pediatric HP, providing a basis for standardized practices.
◦ Intermittent epigastric pain, GIB, and elevated blood amylase are typical “triad” in clinic, and CT angiography and interventional therapy are the gold standard; sometimes, laparoscopic surgery is available.

## 1. Introduction

Gastrointestinal bleeding (GIB) as a common clinical problem endangers the children lives and health, which causes are diverse and require appropriate diagnostic and treatment measures [1–3]. Although techniques such as endoscopic ultrasound, radionuclide scanning, and selective angiography have been widely used, 10% of the patients remain difficult to be accurately diagnosed [2, 3], especially for pediatric hemosuccus pancreaticus (HP), which refers to blood flowing from the pancreatic duct through the duodenal papilla into the upper digestive tract [4]. For HP patients, intermittent abdominal pain, tarry stool, and hematemesis leading to progressively worsening anemia, potentially torrential and life‐threatening acute upper GIB often poses a lethal risk [4–7]. HP rarely happens in pediatrics, lacking clinical attention, and related reports are few case studies; therefore, early diagnosis is significantly difficult, posing challenges to standardized treatment.

This study aims to report a pediatric HP case secondary to chronic pancreatitis with pancreatic pseudocyst and conduct a mini literature review so that to comprehensively elucidate the clinical characteristics and diagnostic and therapeutic management of pediatric HP, as well as the related research progress, which could provide a basis for clinical diagnosis and treatment strategy as well as standardized medical practices.

## 2. Methods

### 2.1. Data Collection

We conducted a retrospective analysis of a pediatric HP case treated in our department, including the general information, treatment, outcome, and two‐year follow‐up.

### 2.2. Literature Search

We developed a narrative literature review based on PubMed, EMbase, the Cochrane Library, Web of Science, and China National Knowledge Infrastructure (CNKI) until April 2025 by scientifically combining the subject terms “hemosuccus pancreaticus,” “pancreatic,” “pancreatitis,” “gastrointestinal,” “hemorrhage,” “bleeding,” “children,” and “pediatrics.” We compared the screening results and then sought the opinions from a third researcher to decide whether to include the literature with disagreements. Studies were included if they met all following: 1) enrolled pediatric patients (≤ 18 years) with HP and 2) English or Chinese reports with comprehensive case records.

### 2.3. Data Extraction

Two investigators independently extracted data of literature using standardized forms, with discrepancies resolved through discussion or a third‐party adjudication. The extracted information included general study information (publication year, country, and sample size), the participant demographics (age and gender), disease features (anamnesis, whether a pancreatic pseudocyst, the vessel responsible for bleeding, and whether occurred a pseudoaneurysm), and treatment (interventional or surgery). The outcomes focused on gastrointestinal hemorrhage or abdominal pain with follow‐up period.

### 2.4. Data Processing

The laboratory test and follow‐up data were all analyzed and visualized using Graphpad Prism (v. 8.1.1) software.

## 3. Results

### 3.1. General Information

The patient was a 13‐year‐old boy, primarily presenting intermittent epigastric pain along with GIB for half a year, who previously suffered from chronic pancreatitis at about one and a half years ago. Imaging examination revealed pancreatic tail cystic lesions (Figure 1A,B), while ECT, gastroscopy, colonoscopy, and twice angiographies showed no definite responsible lesions for hematemesis. When abdominal pain and hematemesis occurred again at the one week of hospitalization, emergency gastroscopy showed fresh blood overflow from duodenal papilla (Figure 1D), and CT angiography (CTA) revealed a pancreatic tail pseudocyst with splenic artery (SpA) involved and occurring pseudoaneurysm (Figure 1C), which suggested HP to be diagnosed.

**FIGURE 1:**
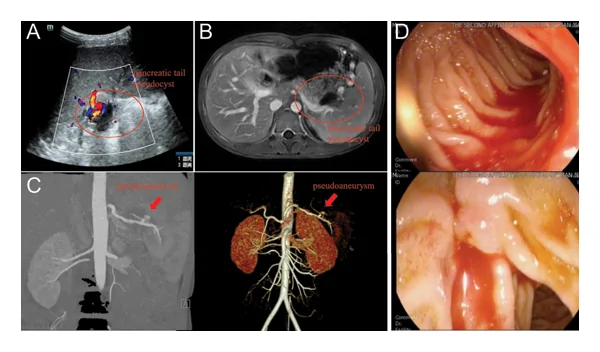
Imaging manifestations of pediatric hemosuccus pancreaticus patient. (A, B) Ultrasound and MRI showed pancreatic tail pseudocyst and multiple tortuous vascular shadows (the red circle indicated). (C) CT angiography revealed a pancreatic tail pseudocyst with splenic artery involved and occurring pseudoaneurysm (the red arrow indicated). (D) The emergency gastroscopy showed fresh blood overflow from duodenal papilla.

### 3.2. Treatment and Outcome

Initially, we performed interventional therapy; when reaching the SpA via right femoral artery approach, the angiography revealed a pseudoaneurysm (Figure 2A1), which was embolized by tissue glue and spring coil (Figure 2A2), and to perform angiography again, there was no aneurysmal blood flow (Figure 2A3). Interestingly, the collateral circulation from the hilus lienis to umbilicus presented left portal hypertension (Figure 2A4). After the intervention, the hemorrhage stopped and laboratory indicators, three‐line blood cells, hemoglobin (Figure 3A), and hematuria amylase (Figure 3B) have temporarily recovered. Even so, two weeks later, the patient experienced hematemesis again, with the hemoglobin dropping to 83 g/L and the blood amylase increased. Therefore, another interventional embolization was performed using a spring coil to occlude the responsible SpA. While re‐examination angiography, the blood flow of the pseudoaneurysm disappeared, with a result of hemoglobin returning to 96 g/L. However, only one week later, the patient experienced one more abdominal pain accompanied by hematemesis, with the hemoglobin dropping to 89 g/L and the blood amylase increased to 268 IU/L.

**FIGURE 2:**
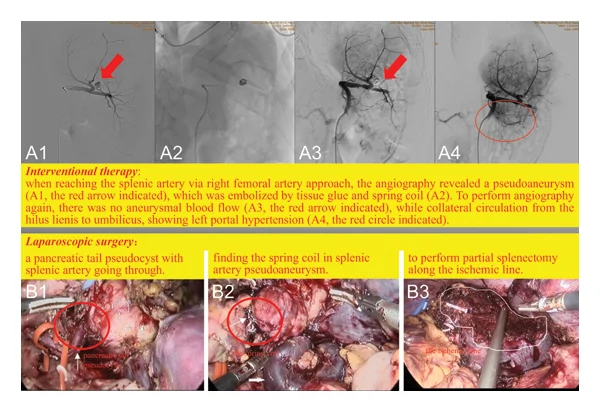
Interventional and Laparoscopic surgery for treating hemosuccus pancreaticus. (A1–A4) Interventional therapy: when reaching the splenic artery via right femoral artery approach, the angiography revealed a pseudoaneurysm (A1, the red arrow indicated), which was embolized by tissue glue and spring coil (A2). To perform angiography again, there was no aneurysmal blood flow (A3, the red arrow indicated), while collateral circulation from the hilus lienis to umbilicus, showing left portal hypertension (A4, the red circle indicated). (B1–B3) Laparoscopic surgery: a pancreatic tail pseudocyst with splenic artery going through (B1, the white arrow indicates the splenic artery); finding the spring coil in splenic artery pseudoaneurysm intraoperatively (B2, the white thin arrow indicates the coil spring, the thick arrow indicates the varicose veins), to perform partial splenectomy along the ischemic line (B3).

**FIGURE 3:**
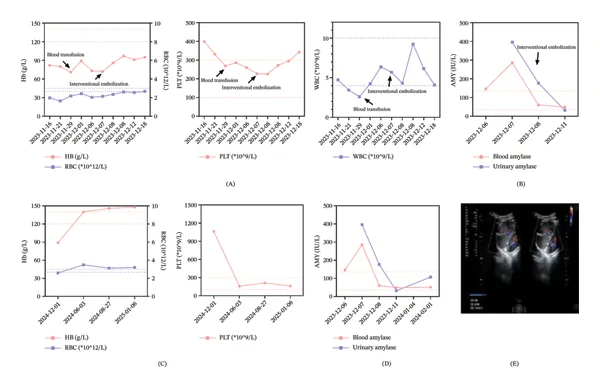
Laboratory indicators after interventional embolization and laparoscopic surgery. After interventional embolization, the laboratory indicators, three‐line blood cells (A), and hematuria amylase (B) have temporarily recovered. The patient was followed up for 1 year after laparoscopic surgery: hemoglobin, red blood cell, platelet (C), and hematuria amylase (D) remained normal. Ultrasound indicated rich blood flow signals with punctate hyperechogenic foci observed in the spleen (E).

After twice interventional therapy, it yielded significant hemostatic effect while appeared prone to recurrence and affected the quality of life of patients; therefore, laparoscopic surgery was performed after a multidisciplinary team (MDT) discussion. Intraoperatively, there existed a pancreatic tail pseudocyst with SpA going through (Figure 2B1) and finding the spring coil in SpA pseudoaneurysm (Figure 2B2). Finally, we performed resection of the pancreatic pseudocyst and SpA pseudoaneurysm, followed by the partial splenectomy along ischemic line excision (Figure 2B3). The pathology was pancreatic pseudocyst and peripheral vascular malformation.

At the first month postoperatively, the patient no longer experienced abdominal pain or hematemesis, with the hemoglobin returning to 125 g/L. When followed up at the 4th, 6th, and 11th months, the hemoglobin, red blood cell, platelet, and hematuria amylase gradually returned to normal (Figure 3C,D), and ultrasound indicated rich blood flow signals with punctate hyperechogenic foci observed in the spleen (Figure 3E). The child no longer complained of any discomfort during the two consecutive years of follow‐up after the laparoscopic surgery.

### 3.3. Mini Literature Review

To comprehensively elucidate the clinical characteristics and management of pediatric HP, we conducted a mini literature review (Table 1). Including this, a total of 10 children were reported, including 8 boys, 1 girl, and 1 unreported gender, with a median age of 11 years (range: 3–18 years). These patients are mainly attributed to peripancreatic pseudoaneurysm ruptured, secondary to acute and chronic pancreatitis, abdominal surgery, or blunt abdominal trauma. Usually, hematemesis and abdominal pain were the common symptoms (Figure 4). Except one case involved a pancreatic pseudocyst that invaded the stomach wall, resulting in gastric wall artery pseudoaneurysm rupture, and other 9 cases attributed to peripancreatic pseudoaneurysm ruptured into the pancreatic duct flowing into duodenum, resulting gastrointestinal hemorrhage. The culprit vessels including: SpA, superior mesenteric artery (SMA), left gastric artery (LGA), and gastroduodenal artery (GDA).

**TABLE 1:** Clinical characteristics and management of HP patients.

Case report	Year	Country	Age	Sex	Anamnesis	Symptoms	Culprit vessels	Diagnosis & treatment	Follow‐up	Outcome
This case	2024	China	13	male	CP	hematemesis, abdominal pain, melena, progressive pallor	SpA	SA: coil embolization laparoscopic surgery	> 2 years	cured
Rai et al.	2024	India	18	male	AP	hematemesis, melena	LGA	EUS, biogel embolization^∗^	3 months	cured
Mohamadnejad et al.	2023	Iran	15	male	TP	hematemesis, abdominal pain	SA	EUS, biogel embolization	6 months	cured
Ekezie et al.	2021	American	12	male	AP	hematemesis	SA	ERCP, SA: coil embolization	unknown	cured
Seetharaman et al.	2021	India	13	male	TP post‐CJ	hematemesis, abdominal pain, melena, progressive pallor	SMA	SA: biogel embolization	9 months	cured
Seetharaman et al.	2021	India	10	male	AP	hematemesis, abdominal pain, melena, progressive pallor	LGA	SA: coil embolization	8 months	cured
Bavanandam et al.	2017	India	3	male	AP post‐CP	hematemesis, abdominal pain, melena, progressive pallor	GDA	SA: coil embolization	> 1 year	cured
Bavanandam et al.	2017	India	7	male	CHCP	hematemesis, abdominal pain, melena,	GDA	SA: coil embolization	> 1 year	cured
Yachha et al.	1996	India	6	unknown	CHCP	hematemesis	GDA	SA: coil embolization	17 months	cured
Ehrensperger	1992	Schweiz	9	female	CHCP	hematemesis, abdominal pain	GWA	percutaneous drainage	> 1 year	controlled

*Note:* CJ, cystojejunostomy; SpA, splenic artery; GDA, gastroduodenal artery; ERCP endoscopic retrograde cholangiopancreatography; EUS, endoscopic ultras.

Abbreviations: AP, acute pancreatitis; CHCP, chronic hereditary calcifying pancreatitis; CP, chronic pancreatitis; GWA, gastric wall artery; LGA, left gastric artery; SA, selective arteriography; SMA, superior mesenteric artery; TP, traumatic pancreatitis.

^∗^When selective arteriography failed, EUS was performed.

**FIGURE 4:**
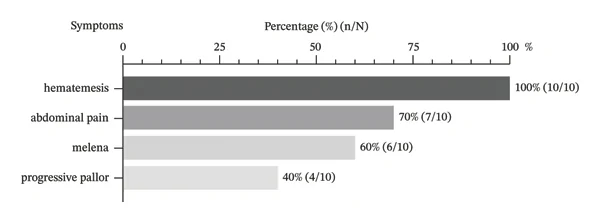
Initial clinical manifestations of HP patients.

For two cases, a pseudoaneurysm was detected through endoscopic ultrasonography (EUS), and under the microscope, there happened overflowing blood from the duodenal papilla, so the HP was diagnosed followed by injecting biological glue into the pseudoaneurysm through EUS. However, endoscopy often missed diagnosis and even requires repeated examination in other patients. This case was ultimately treated with laparoscopic surgery; there were 6 reported cases were diagnosed via CT arteriography and was successfully interventional embolized using coils or biogel. All nine pediatric HP patients no longer experienced abdominal pain or hematemesis with a good prognosis. The patient with the pancreatic pseudocyst rupturing into the gastric wall artery was controlled by conservative treatment, without report about the subsequent treatment and prognosis.

## 4. Discussion

HP is a rare cause in GIB, accounting for 1 in 1500 GIB patients, especially the pediatric HP happens even rarely [5, 8] while has relatively high mortality rate with 7.8% [9] for torrential and life‐threatening acute upper GIB. However, there lacks of standardized diagnosis and treatment experience for pediatric HP in clinical practice. This study aims to comprehensively elucidate the clinical characteristics and management, as well as the related research progress, so that to provide certain theoretical basis for the diagnosis and treatment strategies for HP.

Pediatric HPs are mainly attributed to peripancreatic pseudoaneurysm ruptured into the pancreatic duct to duodenum, commonly secondary to acute and chronic pancreatitis, abdominal surgery, or blunt abdominal trauma [3, 4, 10]. The pancreatic juice leakage or invasive pancreatic pseudocysts can damage the peripancreatic arterial walls, resulting in pseudoaneurysm, meanwhile activate pancreatic enzymes trigger vascular rupture and bleeding [11]. Other rare factors include pancreatic vascular malformations [12], chronic hereditary calcifying pancreatitis, pancreas divisum, and iatrogenic pancreatic injuries (EUS pancreatic puncture or ERCP procedures) [5, 13–15]. The HP children included in this study are all secondary to underlying pancreatic diseases, including various acute and chronic, or traumatic pancreatitis, which seem higher than the proportion reported before (∼60%) [11, 16, 17].

Intermittent epigastric pain, GIB, and elevated blood amylase are common clinical features. Abdominal pain in HP children usually shows a characteristic of “gradually intensifying‐gradually easing,” which occurs when a large amount of blood enters the pancreatic duct, causing local pressure to rise and abdominal pain to intensify, with an increased blood amylase. When the blood is discharged through the Vater’s ampulla, the pancreatic duct returns the normal pressure and the abdominal pain gradually eases [5, 13]. Periodic GIB and black stools lead to progressive worsened anemia, even endangering life sometimes. Due to rarity and the difficulty in early diagnosis, pediatricians should take it into consideration when conducting the differential diagnosis of upper GIB. In clinical practice, in children with a history of pancreatic diseases, intermittent abdominal pain, GIB, and elevated blood amylase, the possibility of HP should be considered.

Abdominal ultrasound and digestive endoscopy are commonly used for these children in clinical practice. Ultrasound can reveal pancreatic pseudocyst combined with pancreatic sinistral portal hypertension (PSPH) appearing tortuous vessels around the splenic hilum and splenomegaly, which serve as indirect signs for diagnosing HP [18]. Endoscopic examination can directly observe the fresh blood flowing out from the duodenal papilla, however often missing diagnosis and even requiring repeated examination, as it can only detect ∼30% of duodenal papilla bleeding [17]. Nevertheless, endoscopic can rule out esophageal and gastric fundus varices, peptic ulcers, erosive gastritis, and other hemorrhagic disorders [2, 19]. This child patient was diagnosed by the second emergency gastroscopy showing fresh blood overflow from duodenal papilla. Endoscopic ultrasound significantly increases the positive detection rate and even enabled endoscopic interventional embolization therapy; however, further exploration is still needed [20–22]. MRI and CT examinations have a relatively high rate of detecting pancreatic pseudocysts, but commonly they can also only provide indirect signs. CTA is recommended for HP diagnosis [5, 13, 23], with a sensitivity of 96%. When angiography revealing hemorrhage from a pancreatic peripancreatic pseudoaneurysm, HP can be diagnosed based on comprehensive endoscopy, ultrasound, or MRI examinations. According to statistical analysis on the cases included in this study, the SpA is most frequently involved peripancreatic vessel, consistent with the previous literature (∼56%) [5], for the closed relationship and it is more likely to be affected by peripancreatic exudation [9]. The next are SpA, SMA, LGA, GDA.

Interventional therapy conducted simultaneously with angiography is the first choice for HP patients, with the overall success rate of 67.0–91.2% [5, 24]. Coils are commonly used interventional embolic materials, which can stimulate the thrombus formatting within the pseudoaneurysm, thereby achieving successful hemostasis [25]. The materials such as biogum, gelatin sponge, or thrombin can also be used. Interventional therapy has advantages such as convenient, with excellent hemostatic effect immediately, however leads to complications including splenic infarction, thrombosis, and recurrent bleeding, especially recurrent bleeding following embolization may reach ∼37%, which can be attributed to the collateral circulation, spring coil erosion and detachment, or pseudoaneurysm recrudescing for the erosion of pseudocysts secondary to chronic pancreatitis [5]. Sometimes, minor lesions are often overlooked and the technical difficulty for small spasmodic vessel procedures often leads the intervention failure. Currently, coverage stent implantation has also been applied, which helps impede the blood perfusion within pseudoaneurysm while ensuring the blood supply of the major vessels and effectively avoiding ischemic injury to the distal spleen [26]. Sometimes, the EUS is alternative, as the latest report describes about EUS‐guided interventional embolization for treating pseudoaneurysms [20].

Patients with failed interventional, ineffective hemostasis and hemodynamic instability, or patients who have concurrent pancreatic pseudocysts, gastric outlet obstruction, abscesses, or pancreatic tumors that require surgical treatment, can be treated with laparoscopic surgery [5]. In most cases, removing the pseudocysts and pseudoaneurysms can achieve hemostasis; however, sometimes it is impossible to carry out, and only to ligate the distal and proximal vessels of the pseudoaneurysm and perform the internal drainage of the pseudocyst. For children with PSPH, based on splenomegaly, splenic hyperfunction, and gastric fundal varices, it is possible to consider whether to perform simultaneous splenectomy and devascularization surgery [27, 28]. For this HP case, after twice interventional therapy, it yielded significant hemostatic effect while appeared prone to recurrence. The recurrence likely resulted from the collateral vessels due to chronic inflammation or the complex anatomy of the pseudoaneurysm. In such cases, repeated embolization increased the risk of ectopic embolization and splenic infarction without guaranteeing hemostasis. Therefore, laparoscopic surgery was selected as a definitive salvage therapy after a MDT discussion to ensure long‐term stability. Intraoperatively, there existed a pancreatic tail pseudocyst with SpA going through. The SpA could not be preserved after repeated assessment, so we severed the SpA and found the spring coil in SpA pseudoaneurysm. Finally, we performed resection of the pancreatic pseudocyst and SpA pseudoaneurysm, followed by the partial splenectomy along ischemic line excision. Laparoscopic partial splenectomy was chosen over a total splenectomy to preserve immune function and avoid overwhelming postsplenectomy infection (OPSI) and thromboembolic complications associated with postsplenectomy thrombocytosis in this adolescent patient. Although the partial splenectomy has been reported to be safe and feasible for children with spleen injury, benign tumor, or hyperfunction, the total splenectomy should not to be minimized when happening intraoperative difficulties in stopping bleeding or a high risk of postoperative bleeding [29–31].

## 5. Conclusion and Limitations

In conclusion, pediatric HPs happen even rarely while early diagnosis is significantly difficult. Intermittent epigastric pain, GIB, and elevated blood amylase are common clinical features, and when happening in children with a history of acute and chronic pancreatitis, the HP should be considered. CTA is recommended for HP diagnosis. Interventional therapy is the first choice, but leading to complications including splenic infarction, thrombosis, and recurrent bleeding. Patients with failed interventional, ineffective hemostasis or hemodynamic instability can be treated with laparoscopic surgery. Sometimes, the EUS is alternative, however needing further study. Generally, the children if diagnosed in time tend to present good prognosis. However, there exist several inherent limitations that must be acknowledged, including the single‐case design, very few published pediatric cases, heterogeneous reporting and follow‐up, potential publication bias, and reliance on adult evidence. The conclusion should also be hypothesis‐generating rather than definitive.

## Author Contributions

Donghao Tian: conceptualization, operation, data curation, investigation, methodology, writing–original draft, and writing–review and editing. Hui Yu, Jialu Fu, Qiang Liu, Yuekui Luo, and Baijun Zheng: methodology, data curation, and investigation. Ya Gao and Donghao Tian: writing–review and editing, project administration, supervision, and funding acquisition.

## Funding

The study was supported by grants from the National Natural Science Foundation of China (Nos: 82300575 and 82170531) and Xi’an Jiaotong University (No: xzy012024111).

## Consent

This study adhered to the principles of the Declaration of Helsinki and was approved by the Medical Ethics Committee of the Second Affiliated Hospital of Xi’an Jiaotong University (No. 122/2024). Informed consent was obtained from the guardians of the pediatric patient, who signed the surgical informed consent form.

## Conflicts of Interest

The authors declare no conflicts of interest.

## Supporting Information

Additional supporting information can be found online in the Supporting Information section.

## Supporting information


**Supporting Information** CARE‐checklist.

## Data Availability

All data relevant to this case report have been included within the manuscript. No additional data are available for review.
