# Supplementary material for: Clinical Management of Pediatric Hemosuccus Pancreaticus: Experience Based on a Case Report and Mini Literature Review
Source: Case Rep Gastrointest Med. 2026 Oct 26;2026:4250947. doi: 10.1155/crgm/4250947 (PMC13698648; doi:10.1155/crgm/4250947)
Supplement: Supplementary file 1 [file CRGM-2026-4250947-s001.pdf]

Checklist adapted from https://www.care-statement.org/checklist		
		**YES**
**Title**	Includes main diagnosis and/or intervention followed by the words “a case report” or “a case series”.	√
**Keywords**	2 to 5 relevant keywords, including “case report” or “case series” as one of them.	√
**Abstract**	Correct length and format (Introduction/Case Presentation/Conclusion).	√
	Introduction: presents the novelty of the case/cases.	√
	Case presentation: includes main symptoms, signs, interventions and outcomes.	√
	Conclusion: includes the main lesson learned by the case/cases.	√
**Introduction**	Provides a brief background of the case referencing the most relevant medical literature.	√
	Includes the CARE statement:<br>- This manuscript was prepared following the CARE guidelines (https://www.care-statement.org).	√
**Case/Cases description**		
* Patient information	Includes demographic data without any identifiers.	√
	Includes the patient’s primary symptoms and relevant history.	√
* Clinical findings	Describes clearly all the relevant physical exam and clinical findings.	√
* Diagnostic assessment	Describes clearly all the relevant diagnostic tests and their key findings.	√
* Interventions	Describes clearly all interventions, medical and/or surgical. Provides key details of all surgical procedures.	√
* Outcomes / Follow-up	Describes the outcomes of all interventions, including any unanticipated events. Describes all relevant findings on follow-up.	√
**Discussion**	Includes a comprehensive discussion of the most relevant literature, including references.	√
	Includes a statement on strengths and limitations of the report.	√
**Conclusion**	Includes the main lesson learned by the presented case/cases.	√
**Statements**	Includes the statements:<br>- Informed consent has been obtained from the patient’s parents or guardian.<br>- All authors attest that they meet the current ICMJE criteria for Authorship.	√
